# Multi-proxy bioarchaeological analysis of skeletal remains shows genetic discontinuity in a Medieval Sicilian community

**DOI:** 10.1098/rsos.240436

**Published:** 2024-07-24

**Authors:** Aurore Monnereau, Alice Ughi, Paola Orecchioni, Richard Hagan, Helen M. Talbot, Efthymia Nikita, Derek Hamilton, Petrus Le Roux, Alessandra Molinari, Martin Carver, Oliver E. Craig, Camilla F. Speller, Michelle M. Alexander, Nathan Wales

**Affiliations:** ^1^ Department of Archaeology, BioArCh, University of York, York YO10 5DD, UK; ^2^ Dipartimento di Storia, Patrimonio Culturale, Formazione e Società, Università degli Studi di Roma Tor Vergata, Rome, Italy; ^3^ Science and Technology in Archaeology and Culture Research Centre, The Cyprus Institute, Nicosia 2121, Cyprus; ^4^ SUERC, University of Glasgow, Rankine Avenue, East Kilbride, Glasgow G75 0QF, UK; ^5^ Department of Geological Sciences, University of Cape Town, Rondebosch, 7701 Cape Town, South Africa; ^6^ Department of Anthropology, University of British Columbia, Vancouver, Canada V6T 1Z1

**Keywords:** ancient DNA, isotope analysis, archaeological science, Sicily, Middle Ages

## Abstract

The medieval period in Sicily was turbulent, involving successive regime changes, from Byzantine (Greek Christian), Aghlabid (Sunni Muslim), Fatimid (Shīʿa Muslim), to Normans and Swabians (Latin Christian). To shed new light on the local implications of regime changes, we conducted a multidisciplinary analysis of 27 individuals buried in adjacent Muslim and Christian cemeteries at the site of Segesta, western Sicily. By combining radiocarbon dating, genome-wide sequencing, stable and radiogenic isotopic data, and archaeological records, we uncover genetic differences between the two communities but find evidence of continuity in other aspects of life. Historical and archaeological evidence shows a Muslim community was present by the 12th century during Norman governance, with the Christian settlement appearing in the 13th century under Swabian governance. A Bayesian analysis of radiocarbon dates from the burials finds the abandonment of the Muslim cemetery likely occurred after the establishment of the Christian cemetery, indicating that individuals of both faiths were present in the area in the first half of the 13th century. The biomolecular results suggest the Christians remained genetically distinct from the Muslim community at Segesta while following a substantially similar diet. This study demonstrates that medieval regime changes had major impacts beyond the political core, leading to demographic changes while economic systems persisted and new social relationships emerged.

## 1. Introduction

Due to its agricultural fertility and strategic position in the central Mediterranean, Sicily has long attracted a diversity of people. During the Middle Ages (5th to 13th centuries CE) Romans, Greeks, Byzantines, Muslims and Latin Christian Northern Europeans competed for control. In 827 CE, the Aghlabid (Sunni Muslim) forces arrived at Mazara from North Africa and by 910 CE had subdued the entire island. From 910 CE, Fatimid Shīʿa Muslims from North Africa sought power in Sicily and in 948 CE, the Fatimid dynasty of the Kalbids took control [1]. They created a prosperous province of the Fatimid Empire with its capital at Palermo from 948–1053 CE. In 1061 CE, Norman Christians led by the Hauteville family invaded Messina and established the kingdom of Sicily under Roger II and his successors from 1130–1189 CE [1,2]. In 1194, Swabians led by the Hohenstaufen dynasty took governance of the kingdom, ruling until the death of Frederick II (*stupor mundi*) in 1250 CE.

While these well-documented histories of changing regimes provide a geo-political context for the period, many of the social and economic impacts are far less discernible. The elites who feature in the chronicles were principally seeking political and religious control through military force, wealth and monumentality, but the effects felt amongst the larger population are far from clear. Historical sources indicate that marriage between Muslims and Christians occurred in both Sicily and Spain, although attempts were made to discourage it [3,4]. For example, in 973 CE during the Fatimid dynasty, Muslim traveller Ibn Hawqal observed widespread acculturation in western Sicily, with mixed marriages between Muslim and Christian individuals [5]. The degree to which interfaith marriages occurred during the later Norman/Swabian period remains a question that requires further investigation. Various parameters could be employed to observe this phenomenon, including genetic evidence and the distinctiveness of the cuisines and diets of these individuals, which could serve as useful indicators of social rules.

**Figure 1.**
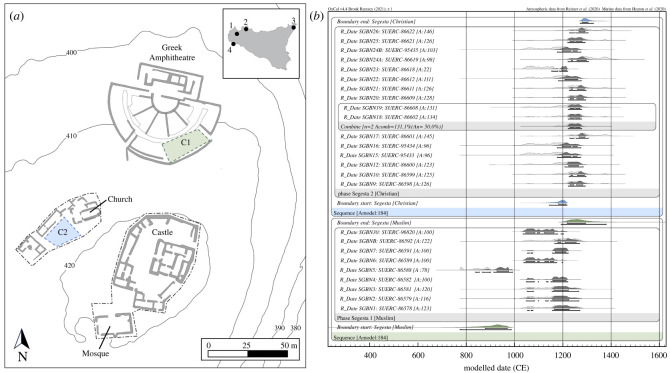
Location and chronology of Segesta cemeteries. (*a*) Site of Segesta indicating the location of the Muslim (C1) and Christian (C2) cemeteries with an inset map showing the island of Sicily with the location of Segesta (1) and the cities mentioned in the text: Palermo (2), Messina (3), and Mazara (4); (*b*) Chronological model of the radiocarbon dates for Muslim and Christian burials at Segesta using OxCal v4.4 and following the methods described in the Supplemental Information.

Human remains provide a direct source of information regarding the lifeways, genetic and cultural affinities of non-elites living through these periods of political turbulence. As such, they provide a powerful, independent source of evidence, enhanced through the application of biomolecular techniques to reveal dietary patterns, residential mobility, genetic diversity and kinship. Using these approaches, here we examine communities of different faiths buried in adjacent cemeteries at Segesta in northwestern Sicily (Trapani Province, figure 1*a*) as it came under Norman and Swabian control during the 12th to 13th centuries CE [6,7]. The Muslim cemetery, situated on the edge of the Greek theatre, consisted of 75 burials, with individuals' faces oriented southeast toward Mecca [6,7]. Approximately 60 m to the southwest, a Christian cemetery was situated on the west side of a church that had been originally constructed circa 1200 CE [8]. The Christian cemetery consisted of multiple burials in 57 stone tombs, with those individuals who were undisturbed lying supine with folded arms [8,9].

By analysing individuals buried under Islamic and Christian rites, we aimed to elucidate the chronological sequence at Segesta and compare aspects of their lifeways, through diet, mobility and genetic ancestry. The correlation between ancestry, diet and faith has rarely been directly examined in historical populations and it is particularly unusual to document the degree of demographic, economic and cultural change during a period of dramatic political transition. To achieve this aim, 27 individuals buried in the Islamic (*n* = 9) and Christian traditions (*n* = 18) were selected for multi-proxy biomolecular analyses (electronic supplementary material, dataset S1: Genetic and radiocarbon analyses; electronic supplementary material, dataset S3: stable isotopes, electronic supplementary material, dataset S4: compound-specific isotope analysis (CSIA)). All of the 27 individuals were analysed for carbon and nitrogen stable isotope analysis to assess potential dietary differences, 17 for strontium and oxygen to assess levels of mobility, as well as 25 for radiocarbon dating to refine the chronology of the cemeteries. Of the nine individuals from the Muslim cemetery and the 18 individuals from the Christian cemetery, eight and 13 were respectively assessed for genome-wide screening to investigate genetic similarity, demographic continuity and potential kinship within and between the two cemeteries. A full description of the sampling is provided in electronic supplementary material, information (electronic supplementary material, dataset S1, dataset S3).

## 2. Material and methods

### 2.1. Radiocarbon dating

Detailed information about the burials and individuals can be found in the electronic supplementary materials (electronic supplementary material (SI) text §1, dataset S1 and dataset S3). Twenty-five of the samples were radiocarbon-dated using accelerator mass spectrometry (AMS) at SUERC, the University of Glasgow. Radiocarbon results were calibrated with the IntCal20 atmospheric curve [10] using OxCal online version 4.4; reservoir effects were applied where appropriate when undertaking Bayesian chronological modelling to examine whether the cemeteries were used at the same time [11,12] (see electronic supplementary material text §2, dataset S1, dataset S5).

### 2.2. aDNA analysis

Ancient DNA analysis was undertaken to examine the genetic diversity and kinship of the Segesta population. Twenty-one individuals from Segesta were sampled for aDNA analysis. The petrous portion of the temporal bone was preferentially selected in order to maximize the recovery of human endogenous DNA. If a petrous was not available, then either a tooth or a long bone was selected [13]. The ancient DNA laboratory experiments were conducted within the ancient genomics laboratory at BioArCh, University of York, UK; detailed methods are presented in the electronic supplementary material, text §3. DNA was extracted from skeletal elements, converted into double-stranded Illumina libraries [14], and sequenced on an Illumina HiSeq platform at the GeoGenetics Sequencing Core in Copenhagen, Denmark and Novogene in Sacramento, California, USA (electronic supplementary material, text §3). Mitochondrial DNA was enriched in two samples with low endogenous DNA content using a myBaits hybridization capture kit (Arbor Biosciences, Ann Arbor, Michigan, USA) [15]. Samples which exhibited the expected patterns of DNA degradation [16] and demonstrated no contamination in the mitochondrial genome [15] and X chromosome [17] were included in further analyses. Using a minimum mapping quality of 30 and base quality of 30, uniparental haplogroups were determined by using HaploGrep [18] and Yleaf [19]. Ancient DNA data from Segesta and Himera [20] were merged with published data for the Human Origins (HO) dataset and archaeological individuals compiled in the Allen Ancient DNA Resource (V50) [21]. To avoid the impact of uncorrected DNA damage, analyses excluded transition sites. A pseudo-haploid approach was implemented for nuclear SNP loci, using the random sampling of one read, and then using EIGENSOFT [22,23] for PCA analysis and ADMIXTURE [24] to infer ancestry components. To estimate affinities among populations, we calculated outgroup-*F*_3_-statistics using ADMIXTOOLS software option ‘qp3pop’ and ‘inbreed:yes’ [25].

### 2.3. Isotope analysis

Full details of stable and radiogenic isotope analysis are presented in the electronic supplementary material, information (electronic supplementary material, text §4 and §5). Bone collagen extraction was carried out according to established protocols, including an additional ultrafiltration step [26,27]. Collagen from adult humans (*n* = 11) and terrestrial and marine fauna (*n* = 21) was selected for compound-specific stable isotope analysis based on the samples with the best preservation. Collagen was prepared for GC-C-IRMS, following hydrolysis to release amino acids. For these, *δ*^15^N measurements were carried out on at least nine individual amino acids using the approach previously described by Soncin *et al*. [28]. Quality control criteria are described in electronic supplementary material text §4 and §5. The *δ*^18^O values of tooth enamel carbonates were determined following the procedure described by Miller *et al*. [29], while tooth enamel was prepared for ^87^Sr/^86^Sr measurements following the procedure described by Leggett *et al*. [30].

## 3. Results and discussion

### 3.1. Chronology, kinship and ancestry

We examined the chronological relationship between the burials at the two cemeteries to establish whether they were drawn from communities living contemporaneously at Segesta. Direct AMS dating of bone collagen from 25 individuals, 9 from the Muslim cemetery and 16 from the Christian cemetery, indicates that the former began before the latter (figure 1*b*; electronic supplementary material, dataset S1). Bayesian chronological modelling estimates the Islamic burials began between *770–990 CE* (*95% probability*; figure 1*b*) and ended by *1190–1380 CE*, while the Christian burials started between *1140–1220 CE* and concluded by *1270–1330 CE*. A formal comparison of the posterior distributions indicates there is a 99.3% probability the dated Christian burial activity began before the end of the Muslim burial activity, from which we conclude that both communities likely resided concurrently for some period of time in the early 13th century. These results complement archaeological evidence that after the arrival of the Christian newcomers, Muslims continued to live nearby. For example, structures found away from the Norman castle at Segesta employed ground plans and building techniques of rough-cut stones bonded with earth, forms used in the superseded Muslim phase at the castle site [31]. In a similar manner, excavations of occupation levels in Area SAS 5 led to the discovery of coinage of both Henry VI (1194–1197) and rebel leader Muhammad Ibn Abbād (c. 1220) [31,32].

We evaluated genetic sex and kinship in the individuals using DNA analysis of the skeletal material. Endogenous human DNA was successfully extracted from 21 of the AMS-dated individuals, accounting for 0.3–42.6% of the obtained sequences (mean = 15.4%) and yielding a depth of coverage on the nuclear genome of 0.00–1.85× (mean = 0.48×; see electronic supplementary material, dataset S1). DNA damage profiles and contamination estimates were consistent with degraded DNA with minimal human contamination (electronic supplementary material, figure S1; dataset S1). Genetic sex determinations [33] were largely in agreement with the osteological assessment of the adult skeletons, except for SGBN9 (electronic supplementary material, dataset S1), who is presumed to have been misidentified osteologically. Genetic sexing of non-adults showed that one child from the Muslim cemetery was genetically male (XY), one child from the Christian cemetery genetically female (XX), and the other seven children buried in the Christian rite were genetically male (XY). To identify biological kinship relationships within and between the cemeteries, we explored uniparental markers (mitochondrial DNA and Y-chromosomal haplotypes) as well first, second, and third-degree relationships through nuclear single nucleotide polymorphisms (SNPs) using READ software [34]. Although a number of shared mitochondrial DNA and Y-chromosomal haplogroups highlighted potential biological relationships within both cemeteries, familial relationships were only confirmed using nuclear DNA within the Christian cemetery. These were identified first-degree relationships (i.e. parents/offspring or siblings) between individuals SGBN18/SGBN19 and SGBN20 (electronic supplementary material, dataset S1)—all male infants interred in Tomb 17, and with similar dates (SGBN18: 1215–1365 cal CE; SGBN19: 1195–1305 cal CE; SGBN20: 1225–1385 cal CE). As the sequences were obtained from three complete petrous bones, the combined genetic and osteological data suggest that SGBN18 and SGBN19 (right and left petrous) were likely to have been the same individual or twins (hereafter jointly analysed as SGBN18_19), whereas SGBN20 was a sibling. No further relationships (i.e. second- or third-degree) were discernible.

We next explored uniparental genetic markers. Most individuals carried mitochondrial DNA haplotypes which are widely distributed across Eurasia; however, SGBN2, a male buried in the Muslim cemetery carried mitochondrial DNA haplotype L3e5 which is primarily found in sub-Saharan Africa (see details in electronic supplementary material, text §3 and dataset S1). The Y-chromosome haplotypes were suggestive of differences between the cemeteries: individuals from the Muslim cemetery carried haplotypes associated with North Africa (E1b-M81 and E1b-M310.1) [35,36] and the Eastern Mediterranean (J2b-M241) [37], while four of the nine individuals buried in the Christian cemetery belonged to haplogroup R1b-M269, a haplogroup which is primarily found today in Western Europe [38].

**Figure 2.**
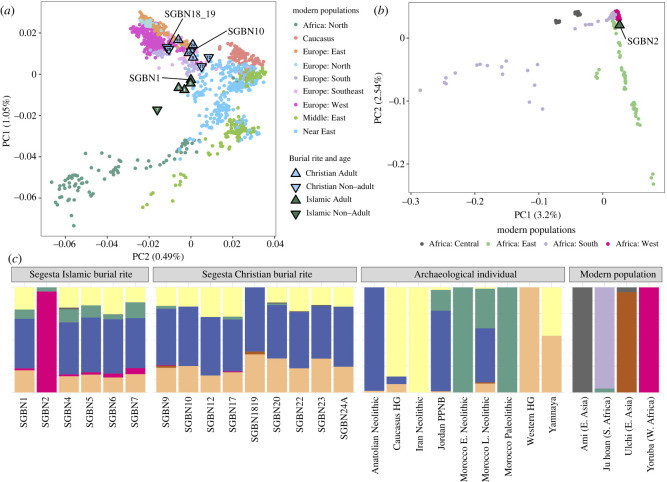
Genetic diversity at Segesta. (*a*) PCA showing Segesta samples (greater than 10 000 transversion SNPs) against a panel of modern Eurasian and North African populations. PC1 is presented on the y-axis as it follows a north-south gradient; (*b*) PCA showing SGBN2 individual against a panel of modern African populations; (*c*) unsupervised ADMIXTURE plot, assuming *K* = 8 ancestral populations.

To undertake a more detailed investigation of ancestry, we analysed the genome-wide data where at least 10 000 transversion SNPs overlapped with the Human Origins SNP panel used in human palaeogenomics [39]. Principal components analysis (PCA) was performed by projecting archaeological individuals onto the diversity of modern populations available in the Allen Ancient DNA Resource (AADR) [21] (electronic supplementary material, dataset S1, dataset S2), using three geographic levels of inquiry. Against a worldwide panel of 141 modern populations, we observed that all Segesta individuals fell within the PCA-space represented by Europe and North Africa, except for SGBN2, who fell within the diversity of sub-Saharan African populations (electronic supplementary material, figure S2). Constricting the analysis to modern Eurasian and North African populations, we observed individuals from the Muslim cemetery showed affinity to one another, plotting between modern populations from Southern Europe, Southeastern Europe, North Africa and the Near East, the latter set including Jewish populations from the Near East and North African (figure 2*a* and electronic supplementary material, figure S3). One individual, SGBN7, plots between Near Eastern populations and modern North African populations. The individuals buried in the Christian cemetery plot separately in PCA-space, generally situated near modern populations from Eastern, Southern, Southeastern and Western Europe. Those results indicate a genetic distinction between the groups, with no examples of individuals in one cemetery having a stronger genetic affinity to those from the other cemetery. While the available assemblage was biased toward males, the number of children from the Christian cemetery provided an opportunity to identify offspring of interfaith unions: none were detected. Continuing at the scale of Eurasia and North Africa, we also evaluated these groups' affinity to Iron Age Sicani individuals from Sicily [20], finding the Christian individuals overlapped the PCA-space occupied by the ancient Sicilian Iron Age individuals (electronic supplementary material, figure S3). When SGBN2 is examined in the context of African populations, we observe the individual plots with groups from West and East Africa (figure 2*b*; electronic supplementary material, figure S4).

The contrast in ancestry between individuals buried in the Islamic tradition compared to those afforded a Christian burial rite was further examined with ADMIXTURE and *F*-statistics. ADMIXTURE analysis indicated that ancient Moroccan and west sub-Saharan ancestries are mainly present within Segesta individuals buried in the Islamic rite (figure 2*c*, electronic supplementary material, dataset S1). We applied an outgroup-*F*_3_-statistic (X, modern/ancient populations: Ju|’hoan_North) to explore population affinities between the ancient Segesta individuals buried under Christian rite, Islamic rite and previously published ancient and modern populations (electronic supplementary material, dataset S2), however, no significant affinities were determined, likely as a result of low numbers of transversions in the dataset (electronic supplementary material, text §3; figure S5–S8).

### 3.2. Lifeways, diet and mobility

Next we set out to determine whether there were any discernible differences in dietary lifeways at Segesta and, if so, whether these correlate with faith and ancestry. The investigation of diet through stable isotope analysis reflects long-term habitual attitudes to food as well as economic practice and social status, all of which may vary culturally in a single locality. The approach has been employed to demonstrate differences in diet between individuals of different faith groups (e.g. Christians and Muslims) in medieval societies [40,41]. For example, differences in the bone collagen stable nitrogen (*δ*^15^N) and carbon (*δ*^13^C) isotope values were noted within the multi-faith community at Gandia on the Mediterranean Iberian coast, dating to the 13th–16th centuries CE, with Muslims consuming a greater amount of marine fish and C_4_ plants (e.g. millet, sorghum and sugarcane) in their diet compared to Christians, perhaps reflecting socially restricted access to resources, such as terrestrial animals and C_3_ plants (cereals, legumes and pulses at this time [41].

**Figure 3.**
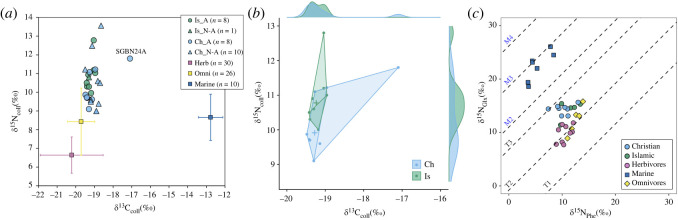
Dietary isotope analysis. (*a*) Scatter plot of bulk stable carbon (*δ*^13^C) and nitrogen (*δ*^15^N) isotope data from humans from Segesta separated by burial rite (Ch = Christian, Is = Islamic), age (A = Adult, N-A = non-adult) including published animals for comparison (Herbivores, Omnivores and Marine fish, mean ±1*σ*) from Mazara [44]; (*b*) Bagplot comparison of *δ*^13^C and *δ*^15^N values for adults from Segesta separated by burial rite, with data distributions indicated on the margins; (*c*) Collagen compound specific stable isotope data plotted as the *δ*^15^N of glutamic acid against the *δ*^15^N of phenylalanine. The trophic position lines (T = terrestrial trophic position, M = marine trophic position) shown are from Naito *et al*. [45] for reference only [see 46].

At Segesta, collagen was extracted for stable isotope analysis from a slightly larger set of human remains compared to those selected for AMS dating and genomic analysis, partly in order to more reliably compare the populations but also due to sample availability. Notably, infant diets may vary more widely and partially reflect the effects of breast-feeding [42]. The petrous bone, preferentially targeted here for enhanced aDNA preservation, may also reflect infant dietary practices due to its relatively lower turn-over rates [43]. Significantly, there were no significant differences between bulk *δ*^15^N and *δ*^13^C of collagen from adults buried under Islamic and Christian traditions (figure 3*a,b*, electronic supplementary material, dataset S3), such as one may expect due to habitual differences in diet. All the individuals bar one (SGBN24A) had diets dominated by C_3_ plants or C_3_ foddered terrestrial animals, with no significant enrichment in collagen ^13^C (i.e. >1‰) compared to comparative values for 10th–13th centuries terrestrial fauna from Mazara [44]. While a lack of C_4_ cereals (millet and sorghum) is expected during this phase in Sicily, as reflected in archaeobotanical assemblages from across the island [47,48], the apparent absence of marine foods is interesting given the site's proximity to the coast (ca. 10 km).

To investigate diet further, we deployed a higher resolution compound-specific approach and measured the *δ*^15^N values of individual amino acids hydrolysed from the collagen of 13 adult individuals: five from Islamic and eight from Christian burials. Amino acids can be traced to dietary sources with greater certainty than bulk bone protein, allowing the contribution of dietary sources to be quantified at greater precision and accuracy [e.g. 28]. Of these, the *δ*^15^N values of phenylalanine (Phe) and glutamic acid (Glu) have been used to better determine trophic position and the degree of aquatic protein consumption [49]. Even using this higher resolution approach, there were no discernible differences between individuals buried according to the different faiths (figure 3*c*); the majority of individuals have Glu/Phe *δ*^15^N spacings (Δ^15^N_Glu-Phe_) indicative of diets dominated by terrestrial plant/animals, with just a single individual (SGBN15, a Christian burial) having greater access to marine foods during their life. Using this approach we were are also able to confirm that the ^13^C enrichment noted in the collagen of SGBN24 most likely derived from the consumption of C_4_ plants, or C4 fed animals, rather than marine foods due to its relatively low estimated trophic position (Δ^15^N_Glu-Phe_ ∼ 5.7‰).

**Figure 4.**
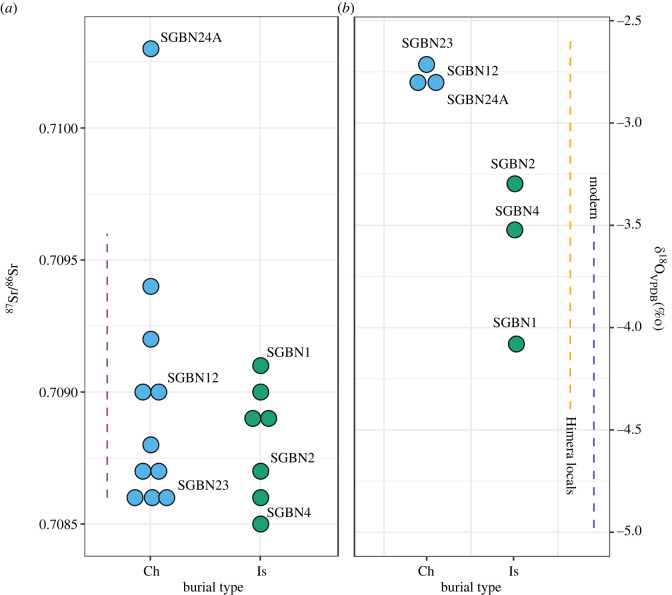
Mobility isotope analysis. Plot of strontium (left) and oxygen (right) data for tooth enamel from humans from Segesta separated by burial rites (Ch = Christian, Is = Islamic). Dashed lines represent predicted local values, for Sr [51], modern drinking water converted to VPDB [52] and published oxygen data from 5th century BCE individuals from Himera [53].

We also obtained information regarding residential mobility by undertaking strontium (Sr) and oxygen (O) isotope analysis of selected individuals where dental samples were available [50] (figure 4). By comparison with the predicted local ranges of both isotopes for Sicily [51–53] there appears to be no convincing evidence of extensive mobility in either community. Individuals from the Christian cemetery have a wider range of Sr isotope ratios, which may indicate, collectively, that they originated from a broader geographic area. One individual buried in the Christian cemetery (SGBN24A) was an Sr outlier and also had a greater proportion of C_4_ foods in their diet, offering convincing evidence of a non-local. With this exception, the remaining Sr ratios reflect values that are within the range of Sicily [51] but also shared in many regions across the Mediterranean and indeed Europe, so some level of migration cannot be ruled out in either period. All *δ*^18^O isotope values are also in keeping with other Southern Mediterranean values and indeed other measurements of other ancient individuals from Sicily [53]. Notably, however, individuals from the Christian cemetery possess higher *δ*^18^O values compared to individuals from the Muslim cemetery, which points to some differentiation in their origin or, perhaps more likely given the lack of difference in their Sr values, access to different sources of drinking water.

### 3.3. Early medieval Segesta: a multi-faith community

The combined historical, archaeological and biomolecular research applied to the populations buried at medieval Segesta has allowed us to elucidate community and interfaith dynamics. Based on the results of our multi-proxy analysis, we propose that if the communities at Segesta were using the respective cemeteries concurrently during the late 12th and early 13th centuries—as seems highly probable from the archaeological finds and dates available—there was little to no interfaith marriage or biological relationships between the individuals we analysed who were buried in Islamic and Christian rites. Christian burials are also apparent throughout the period of Muslim control of Sicily. At Segesta, the genetic data from the period of Swabian control of the island are consistent with a lack of genetic homogeneity between the communities, with closer affinities to modern Europeans for the Christians. Interestingly, the Sr and O data suggests that with one exception, there is little evidence the Christian individuals resided outside Sicily during their childhood, thus they may have descended from immigrants of previous generations. Alternatively, this local signal could result from continuity of a Christian population in Sicily from the Iron Age to the Norman period, however, a larger sample size of individuals from multiple archaeological sites would be needed to explore this hypothesis.

The genetic data from Segesta might appear surprising given the length of time that Muslim and Christian communities co-existed on Sicily. Although Norman governance was in force in Sicily from the mid-11th century and Swabian governance from the end of the 12th century, this is not reflected in the archaeology of the burial rites. Radiocarbon dating at Segesta has shown that burial in the Islamic rite was practised from the 9th century to the 13th, only ceasing after the mid-13th century when many Muslims were deported under Frederick II [1,54,55]. Even if we take into consideration difficulties in distinguishing Arab, Berber populations, who may share considerable ancestry, the majority of individuals afforded Christian burials at Segesta date to over a century following the Norman invasion yet remain clearly distinguishable from those in Islamic burials.

Moreover, the proximity of the burial grounds and settlement areas within the former archaic city implies that Muslims and Christians occupied adjacent spaces and had similar access to markets. This view is supported by the study of personal names and land records in Norman times showing that Muslim and Christian communities coexisted in town and country through the Islamic and Norman period [56]. They resided in different quarters of the town and buried in separate cemeteries, but had social relations [1,5,55]. For instance, in 1184 CE, Spanish Muslim traveller Ibn Jubayr observed instances of intermarriage between Muslims and Christians, primarily unions between Muslim men and Christian women. He also reported that Muslims in the countryside lived together with Christians on their estates and were treated well by them [1,5,55]. Christian women in Palermo dressing like Muslims and speaking fluent Arabic [1,5]. It seems that a social divide between the faiths became ‘unequivocal’ only between Muslims and the later Latin Christians [1] appears to be what is reflected by the genetic results at Segesta.

In contrast to lack of evidence for intermarriage, the carbon and nitrogen isotope data shows that both groups had access to foods with similar isotopic values, with no privileged access to commodities, such as fish or meat, which are easily distinguishable using this approach and have been observed elsewhere [41]. Complementary evidence suggests that these were not just broad dietary similarities, but rather that there were shared culinary practices during this period. In particular, the 12^th^-century ceramic assemblage at Segesta was noted by the excavator to imply a certain hybridity in their use, in which local and Islamic types such as filter jars and hand-made cooking pots occurred with more exotic individual plates and bowls [31]. At a wider scale, emerging evidence from chemical analysis of pottery residues from across Sicily shows continuity in the types of foods prepared across periods of Islamic to Norman and Swabian control [57]. While diet and culinary practices represent two proxies of more complex lifeways, they are nevertheless strongly linked with occupation, location of residence and religious affiliation [58]. In Sicily, although Christian political control discouraged interfaith marriage, our data and historical observations of individuals like Ibn Hawqal and Ibn Gubayr suggest that cultural aspects of life were shared between communities.

## 4. Conclusion

This analysis at the site of Segesta provides a unique glimpse into the interactions between religious communities during the Middle Ages, made possible by the long period in which Muslims and Christians co-existed in Sicily (9–13th centuries CE) and the chance of investigating a site at which Muslim and Christian cemeteries lay adjacent and overlapped chronologically. Funerary archaeology provides powerful statements of tradition and cultural and religious affiliation, but less often reveals the details of everyday life. New insights were achieved here using biomolecular investigations. Isotopic analyses indicated individuals enjoyed a similar diet with little evidence for extensive mobility, however, the genetic analyses indicate that the Muslim and Christian individuals were not only separated by the location of their burials but also by their genetic heritage with no evidence of kinship between the two communities. Based on the assemblage analysed here, our results suggest that the two communities at Segesta could have followed endogamy rules, however, this should not be taken as generally applicable to Medieval Sicily without further analysis of other contemporaneous sites. In this sense Segesta provides a snapshot of the later Swabian period arrivals that has yet to be repeated elsewhere.

## Data Availability

The genetic data files are available in NCBI Project PRJNA1082095. All other data is available within the electronic supplementary information [59].

## References

[1] Metcalfe A. 2003 Muslims and christians in norman sicily: arabic-speakers and the End of islam. London, UK: Routledge.

[2] Lewis B. 2002 Arabs in history. Oxford, UK: Oxford University Press.

[3] Johns J. 2002 Arabic administration in norman sicily: The royal diwan, 1st edn. Cambridge, MA: Cambridge University Press.

[4] Echevarría Arsuaga AM. 2020 Retelling interreligious marriage, from Andalusi Christians to Moriscos. Mediterranean Historical Review **35**, 63-78. (10.1080/09518967.2020.1741232)

[5] Dalli C. 2009 Contriving Coexistence: Muslims and Christians in the Unmaking of Norman Sicily. In Routines of Existence: Time, Life and After Life in Society and Religion (ed. E Brambilla), pp. 30-43. Pisa: Pisa University Press.

[6] Di Salvo R. 2004 I Musulmani della Sicilia occidentale : aspetti antropologici e paleopatologici. Mélanges de l’École française de Rome - Moyen Âge **116**, 389-408.

[7] Fabbri PF. 2001 Segesta. Sepolture islamiche dell'area del teatro (SAS 12; 1995): scavo ed analisi antropologica preliminare. Ann. Sc. Norm. Super. Pisa **6**, 495-501.

[8] Pinna A, Sfligiotti P, Firmati M. 1995 Lo scavo dell'Area 2000 (SAS 2). Ann. Sc. Norm. Super. Pisa **25**, 614-631.

[9] Fabbri PF. 1995 Scavo e studio antropologico delle sepolture medievali del SAS 2. Ann. Sc. Norm. Super. Pisa **25**, 632-661.

[10] Reimer PJ et al*.* 2020 The IntCal20 Northern Hemisphere Radiocarbon Age Calibration Curve (0–55 cal kBP). Radiocarbon **62**, 725-757. (10.1017/RDC.2020.41)

[11] Ramsey CB. 2009 Bayesian Analysis of Radiocarbon Dates. Radiocarbon **51**, 337-360. (10.1017/S0033822200033865)

[12] Ramsey CB. 2017 Methods for Summarizing Radiocarbon Datasets. Radiocarbon **59**, 1809-1833. (10.1017/RDC.2017.108)

[13] Hansen HB, Damgaard PB, Margaryan A, Stenderup J, Lynnerup N, Willerslev E, Allentoft ME. 2017 Comparing Ancient DNA Preservation in Petrous Bone and Tooth Cementum. PLoS ONE **12**, e0170940. (10.1371/journal.pone.0170940) 28129388 PMC5271384

[14] Meyer M, Kircher M. 2010 Illumina sequencing library preparation for highly multiplexed target capture and sequencing. Cold Spring Harb. Protoc. **2010**, db.prot5448. (10.1101/pdb.prot5448) 20516186

[15] Renaud G, Slon V, Duggan AT, Kelso J. 2015 Schmutzi: estimation of contamination and endogenous mitochondrial consensus calling for ancient DNA. Genome Biol. **16**, 224. (10.1186/s13059-015-0776-0) 26458810 PMC4601135

[16] Jónsson H, Ginolhac A, Schubert M, Johnson PLF, Orlando L. 2013 mapDamage2.0: fast approximate Bayesian estimates of ancient DNA damage parameters. Bioinformatics **29**, 1682-1684. (10.1093/bioinformatics/btt193) 23613487 PMC3694634

[17] Korneliussen TS, Albrechtsen A, Nielsen R. 2014 ANGSD: Analysis of Next Generation Sequencing Data. BMC Bioinf. **15**, 356. (10.1186/s12859-014-0356-4) PMC4248462 25420514

[18] Weissensteiner H, Pacher D, Kloss-Brandstätter A, Forer L, Specht G, Bandelt H-J, Kronenberg F, Salas A, Schönherr S. 2016 HaploGrep 2: mitochondrial haplogroup classification in the era of high-throughput sequencing. Nucleic Acids Res. **44**, W58-W63. (10.1093/nar/gkw233) 27084951 PMC4987869

[19] Ralf A, González DM, Zhong K, Kayser M. 2018 Yleaf: Software for Human Y-Chromosomal Haplogroup Inference from Next-Generation Sequencing Data. Mol. Biol. Evol. **35**, 1820. (10.1093/molbev/msy080) 29893912

[20] Reitsema LJ et al*.* 2022 The diverse genetic origins of a Classical period Greek army. Proc. Natl. Acad. Sci. USA **119**, e2205272119. (10.1073/pnas.2205272119) 36191217 PMC9564095

[21] Mallick S, Micco A, Mah M, Ringbauer H, Lazaridis I, Olalde I, Patterson N, Reich D. 2023 The Allen Ancient DNA Resource (AADR): A curated compendium of ancient human genomes. *bioRxiv*, 2023.04.06.535797. (10.1101/2023.04.06.535797) PMC10858950 38341426

[22] Patterson N, Price AL, Reich D. 2006 Population structure and eigenanalysis. PLoS Genet. **2**, e190. (10.1371/journal.pgen.0020190) 17194218 PMC1713260

[23] Price AL, Patterson NJ, Plenge RM, Weinblatt ME, Shadick NA, Reich D. 2006 Principal components analysis corrects for stratification in genome-wide association studies. Nat. Genet. **38**, 904-909. (10.1038/ng1847) 16862161

[24] Alexander DH, Novembre J, Lange K. 2009 Fast model-based estimation of ancestry in unrelated individuals. Genome Res. **19**, 1655-1664. (10.1101/gr.094052.109) 19648217 PMC2752134

[25] Patterson N, Moorjani P, Luo Y, Mallick S, Rohland N, Zhan Y, Genschoreck T, Webster T, Reich D. 2012 Ancient admixture in human history. Genetics **192**, 1065-1093. (10.1534/genetics.112.145037) 22960212 PMC3522152

[26] Longin R. 1971 New method of collagen extraction for radiocarbon dating. Nature **230**, 241-242. (10.1038/230241a0) 4926713

[27] Brown TA, Nelson DE, Vogel JS, Southon JR. 1988 Improved Collagen Extraction by Modified Longin Method. Radiocarbon **30**, 171-177. (10.1017/S0033822200044118)

[28] Soncin S et al*.* 2021 High-resolution dietary reconstruction of victims of the 79 CE Vesuvius eruption at Herculaneum by compound-specific isotope analysis. Sci. Adv. **7**, eabg5791. (10.1126/sciadv.abg5791) 34433561 PMC8386925

[29] Ventresca Miller A, Fernandes R, Janzen A, Nayak A, Swift J, Zech J, Boivin N, Roberts P. 2018 Sampling and Pretreatment of Tooth Enamel Carbonate for Stable Carbon and Oxygen Isotope Analysis. J. Vis. Exp **138**, 58002. (10.3791/58002) PMC6126827 30176003

[30] Leggett S, Rose A, Praet E, Le Roux P. 2021 Multi-tissue and multi-isotope (*δ*^13^C, *δ*^15^N, *δ*^18^O and ^87/86^Sr) data for early medieval human and animal palaeoecology. Ecology **102**, e03349. (10.1002/ecy.3349) 33797749

[31] Molinari A. 1997 Segesta II: il castello e la moschea (scavi 1989–1995). Palermo: Flaccovio Editore.

[32] Nenci G et al. 1991 Segesta: storia della ricerca, Parco e Museo Archeologico, ricognizioni topografiche (1987–1988) e relazione preliminare della campagna di scavo 1989, appendice. Annali della Scuola normale superiore di Pisa, Classe di Lettere e Filosofia **21**, 765-994.

[33] Skoglund P, Storå J, Götherström A, Jakobsson M. 2013 Accurate sex identification of ancient human remains using DNA shotgun sequencing. J. Archaeol. Sci. **40**, 4477-4482. (10.1016/j.jas.2013.07.004)

[34] Monroy Kuhn JM, Jakobsson M, Günther T. 2018 Estimating genetic kin relationships in prehistoric populations. PLoS One **13**, e0195491. (10.1371/journal.pone.0195491) 29684051 PMC5912749

[35] Semino O et al. 2004 Origin, diffusion, and differentiation of Y-chromosome haplogroups E and J: inferences on the neolithization of Europe and later migratory events in the Mediterranean area. Am. J. Hum. Genet. **74**, 1023-1034. (10.1086/386295) 15069642 PMC1181965

[36] Fregel R et al. 2018 Ancient genomes from North Africa evidence prehistoric migrations to the Maghreb from both the Levant and Europe. Proc. Natl Acad. Sci. USA **115**, 6774-6779. (10.1073/pnas.1800851115) 29895688 PMC6042094

[37] Thangaraj K et al. 2010 The influence of natural barriers in shaping the genetic structure of Maharashtra populations. PLoS One **5**, e15283. (10.1371/journal.pone.0015283) 21187967 PMC3004917

[38] Balaresque P et al. 2010 A predominantly neolithic origin for European paternal lineages. PLoS Biol. **8**, e1000285. (10.1371/journal.pbio.1000285) 20087410 PMC2799514

[39] Mathieson I et al. 2018 The genomic history of southeastern Europe. Nature **555**, 197-203. (10.1038/nature25778) 29466330 PMC6091220

[40] Alexander MM, Gutiérrez A, Millard AR, Richards MP, Gerrard CM. 2019 Economic and socio-cultural consequences of changing political rule on human and faunal diets in medieval Valencia (c. fifth–fifteenth century AD) as evidenced by stable isotopes. Archaeol. Anthropol. Sci. **11**, 3875-3893. (10.1007/s12520-019-00810-x)

[41] Alexander MM, Gerrard CM, Gutiérrez A, Millard AR. 2015 Diet, society, and economy in late medieval Spain: Stable isotope evidence from Muslims and Christians from Gandía, Valencia. Am. J. Phys. Anthropol. **156**, 263-273. (10.1002/ajpa.22647) 25351146 PMC4303993

[42] Bourbou C, Fuller BT, Garvie-Lok SJ, Richards MP. 2013 Nursing mothers and feeding bottles: reconstructing breastfeeding and weaning patterns in Greek Byzantine populations (6th–15th centuries AD) using carbon and nitrogen stable isotope ratios. J. Archaeol. Sci. **40**, 3903-3913. (10.1016/j.jas.2013.04.020)

[43] Jørkov MLS, Heinemeier J, Lynnerup N. 2009 The petrous bone–a new sampling site for identifying early dietary patterns in stable isotopic studies. Am. J. Phys. Anthropol. **138**, 199-209. (10.1002/ajpa.20919) 18773469

[44] Ughi A, Alexander M. 2021 Stable isotope analysis of animal remains from Mazara. In Mazara/māzar: nel ventre della città medievale (secoli VII-XV). Edizione critica degli scavi (1997) in via tenente gaspare romano (eds A Molinari, A Meo), pp. 557-566. All'Insegna del Giglio.

[45] Naito YI, Honch NV, Chikaraishi Y, Ohkouchi N, Yoneda M. 2010 Quantitative evaluation of marine protein contribution in ancient diets based on nitrogen isotope ratios of individual amino acids in bone collagen: an investigation at the Kitakogane Jomon site. Am. J. Phys. Anthropol. **143**, 31-40. (10.1002/ajpa.21287) 20333711

[46] O'Connell TC, Collins MJ. 2018 Comment on ‘Ecological niche of Neanderthals from Spy Cave revealed by nitrogen isotopes of individual amino acids in collagen’ [J. Hum. Evol. 93 (2016) 82–90]. J. Hum. Evol. **117**, 53-55. (10.1016/j.jhevol.2017.05.006) 28602430

[47] Fiorentino G, Porta M, Primavera M, Sellitto A. 2021 Mazara tra innovazione e continuità: il contributo dell'archeobotanica alla ricostrizione dei paesaggi, dei sistemi agricoli e delle abitudini alimentari tra periodo Bizantino ed Età Moderna. In Mazara/māzar: nel ventre della città medievale (secoli VII-XV). edizione critica degli scavi (1997) in via tenente gaspare romano (eds A Molinari, A Meo), pp. 567-591. All'Insegna del Giglio.

[48] Castrorao Barba A, Speciale C, Miccichè R, Pisciotta F, Nero CA, Marino P, Bazan G. 2023 The Sicilian Countryside in the Early Middle Ages: Human–Environment Interactions at Contrada Castro. Environ. Archaeol. **28**, 240-255. (10.1080/14614103.2021.1911768)

[49] Naito YI, Bocherens H, Chikaraishi Y, Drucker DG, Wißing C, Yoneda M, Ohkouchi N. 2016 An overview of methods used for the detection of aquatic resource consumption by humans: Compound-specific delta N-15 analysis of amino acids in archaeological materials. Journal of Archaeological Science: Reports **6**, 720-732. (10.1016/j.jasrep.2015.11.025)

[50] Nikita E, Alexander M, Cox S, Radini A, Le Roux P, Chaouali M, Fenwick C. 2023 Isotopic evidence for human mobility in late antique Bulla Regia (Tunisia). J Archaeol Sci Rep **47**, 103816. (10.1016/j.jasrep.2022.103816) 36998714 PMC10041345

[51] Lugli F, Cipriani A, Bruno L, Ronchetti F, Cavazzuti C, Benazzi S. 2022 A strontium isoscape of Italy for provenance studies. Chem. Geol. **587**, 120624. (10.1016/j.chemgeo.2021.120624)

[52] International Atomic Energy Agency, World Meteorological Organization. 2022 Global Network of Isotopes in Precipitation. The GNIP Database. See https://www.iaea.org/services/networks/gnip.

[53] Reinberger KL, Reitsema LJ, Kyle B, Vassallo S, Kamenov G, Krigbaum J. 2021 Isotopic evidence for geographic heterogeneity in Ancient Greek military forces. PLoS ONE **16**, e0248803. (10.1371/journal.pone.0248803) 33979334 PMC8115791

[54] Bagnera A, Pezzini E. 2004 I cimiteri di rito musulmano nella Sicilia medievale. Dati e problemi. MEFRM: Mélanges de l’École française de Rome: Moyen Âge **161**, 231-302. (10.1400/13434)

[55] Metcalfe A. 2019 Before the Normans: Identity and societal formation in Muslim Sicily. In Sicily, heritage of the world (eds D Booms, PJ Higgs), pp. 102-119. London: British Museum.

[56] Johns J. 2002 Sulla condizione dei musulmani di Corleone sotto il dominio normanno nel XII secolo. In Atti del I Congresso Internazionale di Archeologia della Sicilia Bizantina, pp. 275-294. Palermo, Italy: Istituto Siciliano di Sudi Bizantini e Neoellenici, Palermo.

[57] Lundy J et al. 2023 Cuisine in transition? Organic residue analysis of domestic containers from 9th-14th century Sicily. R. Soc. Open Sci. **10**, 221305. (10.1098/rsos.221305) 36908986 PMC9993051

[58] Hastorf CA. 2016 The social archaeology of food: thinking about eating from prehistory to the present. Cambridge, UK: Cambridge University Press.

[59] Monnereau A et al. 2024 Multi-proxy bioarchaeological analysis of skeletal remains shows genetic discontinuity in a medieval sicilian community. *Figshare*. (10.6084/m9.figshare.c.7313547) PMC11265863 39050717

